# High seroprevalence and age-associated dynamics of bluetongue and epizootic hemorrhagic disease viruses in North American bison (*Bison bison*)

**DOI:** 10.3389/fvets.2025.1565805

**Published:** 2025-08-20

**Authors:** Catherine Krus, Ian Zander, Tyler J. Sherman, Courtney Maichak, Danielle E. Buttke, Lee Jones, Christie Mayo

**Affiliations:** ^1^Department of Clinical Sciences, Colorado State University, Fort Collins, CO, United States; ^2^Department of Microbiology, Immunology, and Pathology, Colorado State University, Fort Collins, CO, United States; ^3^Department of Environmental and Radiological Health Sciences, Colorado State University, Fort Collins, CO, United States; ^4^Biological Resources Division, National Park Service, Fort Collins, CO, United States; ^5^Natural Resource Program Center—Wildlife Health, United States Fish and Wildlife Service, Bozeman, MT, United States

**Keywords:** bluetongue, Epizootic Hemorrhagic Disease, bison, cross-sectional, One Health, ruminants, *Orbivirus*, *Culicoides*

## Abstract

Bluetongue virus (BTV) and epizootic hemorrhagic disease virus (EHDV) are two viruses belonging to the genus *Orbivirus* that are transmitted via insect vector, the *Culicoides* biting midge, causing disease in domestic and wild ruminants. These infections can lead to significant morbidity, mortality, and production losses in livestock, with economic consequences for cattle and sheep industries. Despite their growing impact due to environmental and anthropogenic changes, little is known of the prevalence of these viruses in North American bison (*Bison bison*). We present the first cross-sectional survey of BTV and EHDV in North American bison, with samples collected from 287 animals across 9 herds in 7 U.S. states from September to November 2023. Using competitive enzyme-linked immunosorbent assays (cELISA), we detected seroprevalence rates of 56.5% for BTV and 57.5% for EHDV. We found higher seroprevalence in North American bison compared to reports in European bison populations, suggesting that bison could potentially serve as incidental hosts of orbiviruses during key transmission periods; however, their role in virus transmission remains uncertain and warrants further investigation, particularly regarding the duration of viremia, potential amplification capacity, and year-to-year variability in PCR positivity. Logistic regression analysis revealed age as a significant predictor for both BTV (OR: 1.15, CI: 1.05–1.26, *p*: 0.006) and EHDV (OR: 1.16, CI: 1.06–1.28, *p*: 0.0014) seropositivity. PCR amplification identified circulating BTV serotypes 6, 11, 13, 17. Additionally, age was negatively associated with PCR positivity for both BTV (OR: 0.70, CI: 0.53–0.93, *p*: 0.014) and EHDV (OR: 0.56, CI: 0.33–0.93, *p*: 0.024), suggesting a decline in detectable viremia with increasing age. Although complex environmental and epidemiological factors likely play a role, this trend may be due to older animals having experienced more vector seasons, thereby increasing their cumulative exposure and subsequent immunity to these viruses over time. The significant age-associated dynamics reveal the importance of considering life stage in disease surveillance and management. Our study also highlights the importance of integrating bison into future vector-borne disease research and control strategies to mitigate risks to livestock, wildlife, and ecosystem health.

## Introduction

Shaped by climate change, globalization, and anthropogenic activities such as farming, land-use modifications, and trade, vector-borne viruses of ruminants (often referred to as arboviruses despite the term having no taxonomic significance) have undergone significant shifts in their epidemiology over the past several decades (1–3) These shifts have contributed to the re-emergence and geographic spread of many arboviruses, including the economically significant bluetongue (BT) and epizootic hemorrhagic disease (EHD).

Bluetongue Virus (BTV) and Epizootic Hemorrhagic Disease Virus (EHDV) are classified in the genus *Orbivirus* and are transmitted by various species of *Culicoides* midges (4, 5). In the genus *Culicodies*, females require a blood meal for ovarian maturation and egg production (6). When a *Culicodies* midge feeds on an *Orbivirus*-infected host, the virus undergoes an extrinsic incubation period of 7 to 14 days, depending on environmental factors like temperature (5). During this time, the virus must overcome physiological barriers in the midgut before disseminating to the salivary glands, where it replicates unchecked and becomes transmissible to a new host during subsequent blood meals (5, 7). Historically, these viruses were confined to tropical and subtropical regions of Africa, but have since expanded into more temperate zones, including Europe, the Americas, and Asia, with projections indicating continued geographic expansion (8–11).

Bluetongue Virus consists of 29 described serotypes, 27–29 of which are putative and capable of segment reassortment (12–15). This contributes to its genetic diversity and complicates vaccine development (16). The clinical manifestations of BTV and EHDV infections vary depending on the host species and virus serotype but can include fever, hemorrhage, abortion, oral ulceration, and edema, with outcomes ranging from subclinical to severe (17–20). These diseases impose significant economic costs through decreased productivity, direct veterinary expenses, and the implementation of control measures (21–23).

While extensive research on BT and EHD has focused on domestic livestock, the epidemiology of these diseases in bison remain poorly understood, with only a handful of studies specifically exploring these diseases in North American or European bison (18, 24–27). As an iconic species of ecological, economic, and cultural importance, bison are uniquely positioned at the interface between domestic livestock and wildlife, with many states listing them under dual classifications, highlighting the critical importance of their health status in understanding disease dynamics (28–31).

This gap is particularly concerning given the unknown potential for bison to facilitate the maintenance and vector-mediated transmission of these viruses to co-grazing domestic livestock and other wildlife. To address this, our study investigated the seroprevalence of BTV and EHDV antibodies in range-limited, minimally managed (handled yearly or biennially) North American bison, identified circulating BTV serotypes via RT-qPCR; and examined age-associated dynamics in correlation to current and past exposure. These findings offer valuable insight into the epidemiology of BTV and EHDV in bison and emphasize the importance of integrated surveillance and control measures to mitigate the broader impact of vector-borne diseases on diverse ruminant populations and ecosystems.

## Materials and methods

### Sample collection

**Figure 1:**
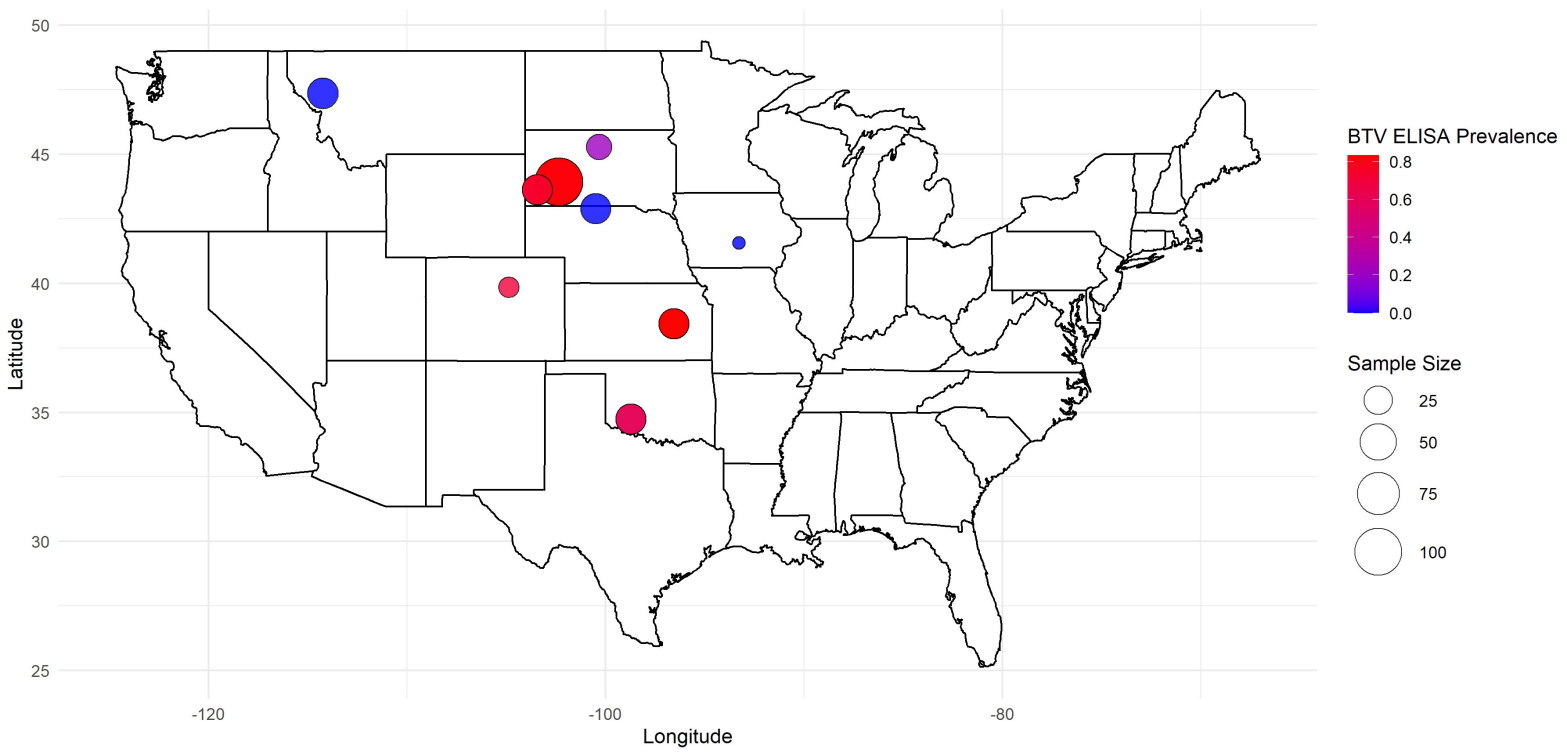
Geographic distribution and herd size of North American bison with associated BTV seroprevalence in a 2023 cross-sectional study on Bluetongue Virus and Epizootic Hemorrhagic Disease.

Samples from a total of 287 North American bison (285 serum, 216 whole blood, with 214 paired) were collected from nine herds in seven states (Table 1, Figure 1). The states included in this study (CO, IA, KS, MT, NE, OK, SD) encompass regions within the historical range of North American plains bison and include areas where significant bison populations persist today. While comprehensive data on private bison herds remain limited, these states are home to several Department of the Interior (DOI) conservation herds that serve as an essential resource for bison conservation and management, along with a few conservation herds managed similarly by other agencies and organizations (32). Sampling was conducted opportunistically during routine management-assoicated capture and handling events scheduled between August 31st and November 13th, 2023, with individuals sampled based on accessibility and availability. All sampling procedures were performed in coordination with herd managers, veterinary personnel, and wildlife biologists to minimize stress and ensure activities met currently accepted professional standards of animal welfare as identified by each agency or managing organization. Serum samples were extracted through centrifugation in the field prior to storage alongside K3EDTA preserved whole blood samples in coolers with ice packs during transport to the Colorado State University Veterinary Diagnostic Laboratory for further diagnostic processing.

**Table 1:** Geographic distribution, seroprevalence, PCR-positivity, and serotype identification of orbiviruses in a 2023 cross-sectional survey of North American bison herds.

**State**	Serum^*^	Whole blood^*^	**Total animals sampled**	**BTV ELISA + (%)**	**EHDV ELISA + (%)**	**EHDV PCR + (n)**	**BTV PCR + (n)**	BTV serotypes^**^
CO	10	10	10	70.0	70.0	0	3	N/A
IA	5	5	5	0.0	20.0	1	1	N/A
KS	30	29	30	83.3	86.7	2	5	6,13
MT	31	31	31	0.0	3.2	0	0	-
NE	30	30	30	0.0	6.7	1	0	-
OK	30	30	30	60.0	53.3	2	7	11,13,13,13
SD	30	31	32	80.0	76.7	0	0	-
SD	101	50	101	82.2	80.2	4	5	6,11,17,17
SD	18	0	18	22.2	33.3	-	-	-

* Total serum and whole blood samples collected per herd.

** BTV serotypes identified using PCR serotyping. Some positive samples could not be successfully serotyped.

N/A was placed in locations where serotypes could not be identified via RT-qPCR.

### ELISA testing for BTV and EHDV antibodies

Competitive enzyme-linked immunosorbent assays (cELISAs) were conducted to detect antibodies against Bluetongue Virus and Epizootic Hemorrhagic Disease Virus using the Bluetongue Virus Antibody Test Kit (VMRD, Washington, US) and EHD Virus Antibody Test Kit (ID Screen, France) respectively. Plates with antigen-coated wells were incubated with serum samples and controls that were supplied by the kit, washed, and sequentially treated with substrate and stop solutions following the manufacturers' protocols. Optical densities were read on a plate reader (BioTek Instruments Inc., Vermont, US) at 630 nm for BTV and 450 nm for EHDV. Final classifications were based on kit validation criteria provided by the manufacturer.

For BTV, samples with a mean percent inhibition (%I) ≥60% were classified as positive. For EHDV, samples with a percent sample-to-negative (%S/N) ratio ≤ 30% were classified as positive, while values of 30% < %S/N < 40% were retested to confirm results.

### RNA extraction

RNA Extraction RNA was extracted from whole blood samples using the MagMAX™ Pathogen RNA/DNA Kit (ThermoFisher, Massachusetts, USA) on a KingFisher 96 automated system according to the manufacturer's protocol. Extracted RNA was stored at −80°C until analysis.

### Duplex RT-qPCR

Bluetongue Virus and Epizootic Hemorrhagic Disease Virus RNA were simultaneously detected using the SuperScript™ III Platinum™ One-Step qRT-PCR kit on an Applied Biosystems™ 7500 Real-Time PCR System (ThermoFisher, Massachusetts, USA). Reactions were conducted in a single tube with 25 μL reaction volumes containing primers and probes targeting conserved regions of BTV and EHDV genomes (33–35). 5 uL of extracted nucleic acids were first denatured at 95°C for 5 mins prior to the addition of the reaction master mix. Thermal cycling conditions then included a reverse transcription step (48°C for 30 min), initial denaturation (95°C for 2 min), and 40 amplification cycles (95°C for 15 s, 56°C for 30 s, 72°C for 30 s). Negative and positive controls were included in each run to validate results. Cycle thresholds (Ct) were determined at 10% of the amplification plateau of the positive amplification control (PAC).

### RT-qPCR BTV serotyping

Samples previously confirmed positive for BTV via RT-qPCR were tested for specific serotypes using RT-qPCR with serotype-specific primers for BTV-2w, BTV-3, BTV-6, BTV-10, BTV-11, BTV-13, and BTV-17 (36). Reactions were performed using the SuperScript™ III Platinum™ One-Step qRT-PCR System, as described above. Thermal cycling conditions then included a reverse transcription step (48°C for 30 min), initial denaturation (95°C for 2 min), and 40 amplification cycles (95°C for 15 s, 56°C for 30 s, 72°C for 30 s). Positive and negative controls were included for quality assurance. Amplification was performed using the Applied Biosystems 7500 Real-Time PCR System. Ct thresholds were determined at 10% of the amplification plateau of the positive amplification control (PAC).

### Data analysis

Data analysis was conducted in R (v4.3.0) using the tidyverse package for data preprocessing and visualization. We obtained weather data from Open-Meteo, which provides meteorological data through numerical weather prediction models. Logistic regression models were used to evaluate associations between demographics and meteorological data (e.g., age, sex, herd demographics, land area, and weather conditions) and outcomes (EHDV/BTV serostatus and RT-qPCR status). Predictors were selected based on biological relevance and exploratory analysis.

Models were optimized using the Akaike Information Criterion (AIC) to balance fit and complexity. To assess multicollinearity among predictor variables, Variance Inflation Factors (VIFs) were calculated using the car package in R. Odds ratios (OR) with 95% confidence intervals (CI) were calculated to assess the strength of associations. Visualizations, including probability curves with confidence intervals, were generated using ggplot2. Effect sizes for significant predictors were highlighted to enhance interpretability.

## Results

Antibodies against BTV were detected in 56.5% (161/285) of bison sera using cELISA, with EHDV seroprevalence at 57.5% (164/285), and 45.3% (129/285) testing positive for antibodies to both viruses. Among the 9 tested herds, BTV seroprevalence ranged from 0% (MT and NE herds) to 83.3% (KS herd), while EHDV seroprevalence ranged from 3.2% (MT herd) to 86.7% (KS herd) (Table 1).

RT-qPCR-based serotyping revealed the presence of multiple circulating BTV serotypes, including BTV-6, BTV-11, BTV-13, and BTV-17. These serotypes were detected in herds from South Dakota, Kansas, and Oklahoma (Table 1). BTV and EHDV RT-qPCR positivity were lower than antibody detection, with BTV RT-qPCR positivity ranging from 0% to 7% and EHDV RT-qPCR positivity between 0% and 20%.

Variation in seroprevalence and RT-qPCR positivity among herds highlighted potential influences of herd size, geography, and local environmental factors. However, broader herd-level logistic regression models incorporating factors such as herd composition, land area, and climatic variables (temperature, precipitation, windspeed) did not identify statistically significant predictors of seropositivity or RT-qPCR positivity.

**Figure 2:**
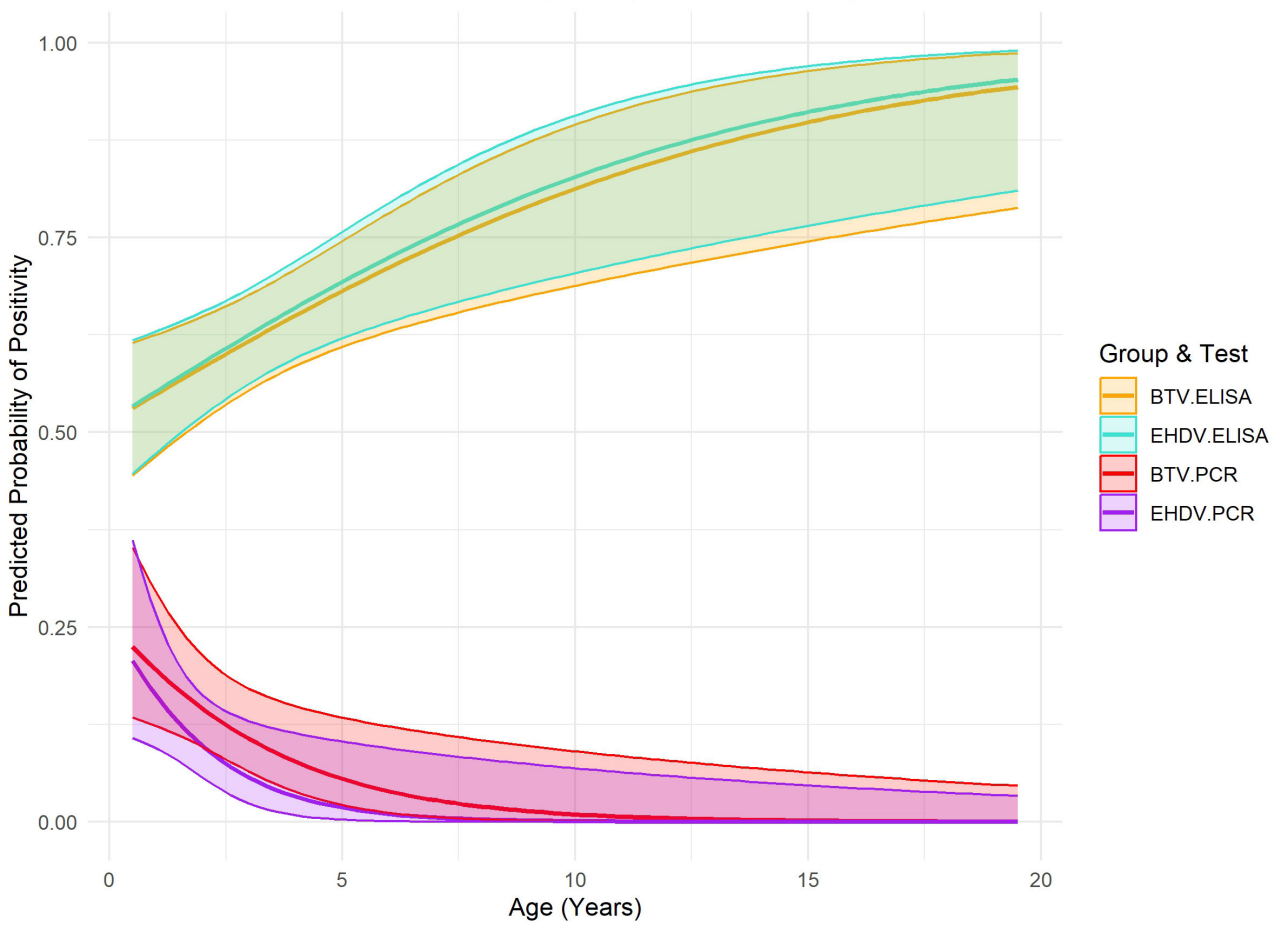
Predicted probability of positivity (ELISA and PCR) for Bluetongue Virus and Epizootic Hemorrhagic Disease Virus by age in bison from a 2023 cross-sectional study, including 95% confidence intervals.

Variables with high multicollinearity (VIF > 10) were excluded from the final analysis to improve model stability and interpretation. Logistic regression analysis identified significant relationships between age and seropositivity for both viruses (Figure 2). For BTV, each additional year of age increased the odds of seropositivity by 15% (OR: 1.15, CI: 1.05–1.26, *p*: 0.006). Similarly, for EHDV, each additional year of age was associated with a 16% increase in the odds of seropositivity (OR: 1.16, CI: 1.06–1.28, *p*: 0.0014). Age was also significantly negatively associated with RT-qPCR positivity for both viruses. For BTV, animals were less likely to be RT-qPCR positive (OR: 0.70, CI: 0.53–0.93, *p*: 0.014) as they aged, and the same pattern was observed for EHDV (OR: 0.56, CI: 0.33–0.93, *p*: 0.024). No significant associations with sex were observed for either seropositivity or RT-qPCR positivity (Table 2). No significance was associated with daily precipitation or 5-day average temperature and RT-qPCR positivity (Table 3).

**Table 2:** Logistic regression analysis of sex as a function of BTV and EHDV test results from a BTV/EHDV cross-sectional survey in bison.

**Dependent variable**	**Independent variable**	**Estimate (β)**	**Std. error**	***p*-value**
BTV PCR	Sex	0.8245	0.4825	0.08747
EHDV PCR	Sex	0.9327	0.6814	0.171021
BTV ELISA	Sex	0.1089	0.4082	0.789565
EHDV ELISA	Sex	−0.3441	0.4075	0.398326

No significance was noted.

**Table 3:** Weather variables and BTV/EHDV PCR results from logistic regression modeling for a BTV/EHDV cross-sectional survey in bison.

	**EHDV PCR (β)**	**EHDV 95% CI**	**BTV PCR (β)**	**BTV 95% CI**
Daily precipitation	0.090	−0.361, 0.541	0.105	−0.230, 0.439
5-day avg temp.	−0.228	−1.286, 0.830	−0.060	−0.697, 0.576

No significance was noted.

## Discussion

This study identified notably higher seroprevalence rates for BTV and EHDV in North American bison compared to previously reported values of 12.83% and 22.1% for BTV in European bison (*Bison bonasus*) populations (24, 25). While comparisons with European bison seroprevalence are valuable, it is crucial to recognize fundamental differences in BTV and EHDV transmission dynamics between North America and Europe, including differences in habitat and climate. In the United States, these viruses persist in endemic cycles, sustained by continuous vector activity in some geographic regions, whereas in Europe, outbreaks tend to be sporadic and are more tightly controlled through mitigation efforts (37–39). Additionally, European bison and North American bison are distinct species with potentially differing susceptibilities, immune responses, and ecological interactions with vectors. While EHD outbreaks in North America are commonly associated with high mortality in white-tailed deer, bison are generally considered incidental hosts and often remain asymptomatic, as noted during a natural outbreak in a captive facility (40). However, the 2012 EHDV epidemic in the United States revealed that morbidity in bison could reach as high as 7%, highlighting the potential risk to this species under certain conditions (20). Combined with the observed high seroprevalence in the present study, these findings suggest that bison could potentially contribute to *Orbivirus* transmission as incidental hosts, particularly during the first 2 years of life when they are more likely to be infective. As with European red deer, which do not appear to maintain BTV in France, it remains uncertain whether bison can sustain transmission cycles of if they are spillover hosts (41). Bison's role in transmission ecology is likely limited compared to species with prolonged periods of viremia, such as noted with EHDV in white-tailed deer (42). Further research into their role in *Orbivirus* ecology and implications for disease transmission dynamics is warranted.

There are many aspects that further complicate disease dynamics in bison, including BTV/EHDV serotype co-circulation and reassortment. Immunity to one serotype does not often provide effective protection against another (43–45). Additionally, BTV and EHDV exhibit strain-dependent variations in virulence, with different strains of the same serotype causing varying levels of clinical disease (46, 47). The detection of multiple BTV serotypes in the present study highlights the complexities associated with serotype-specific immunity and the potential for novel serotype introductions to precipitate outbreaks. During the BTV-8 novel outbreak in Europe (2006–2008), BTV-8 caused the deaths of 10 of 33 bison in a German breeding center along with up to a 40% morbidity and 20% mortality in European zoos (19, 25). Reported clinical signs included lethargy, fever, mouth ulcers, drooling, difficulty eating, conjunctivitis, corneal edema, respiratory difficulty, lameness, inflammation of the coronary band, and sudden death (19). Notably, North American bison experimentally infected with BTV-11 developed detectable antibodies without exhibiting clinical signs (18). This finding highlights the ability of bison to mount an immune response to a specific serotype, potentially reducing clinical disease severity in subsequent exposures to the same serotype. The age-dependent dynamics observed in the current study may also reflect the accumulation of partial immunity in older animals due to prior exposures, which could mitigate the impact of subsequent infections. However, the introduction of a new serotype, to which the population has no prior exposure, can still result in high morbidity and mortality, as evidenced by the recent European outbreaks (39, 48). These events emphasize how exposure to novel serotypes can lead to significant disease outbreaks, particularly in populations lacking prior immunity.

Geographic variability also plays a critical role in *Orbivirus* transmission (42, 49). The wide range of seroprevalence observed between sites in the current study highlights the role of localized environmental factors and vector habitats in shaping *Orbivirus* transmission dynamics. Bison wallows, for example, serve as temporary breeding sites for *Culicoides* spp., with species like *Culicoides sonorensis* favoring active wallows enriched by bison activity (27). These findings suggest that landscape features and host behaviors can create focal points for vector-host interactions, influencing disease transmission at a local level. Additionally, the 2012 EHD outbreak in the U.S. demonstrated geographic clustering, with most cases in cattle and bison occurring in Nebraska, South Dakota, and Iowa (20). Similarly, cattle studies have reported region-specific seroprevalence rates, emphasizing the impact of geographic and ecological factors on disease exposure risk (22).

In a broader context, climate change is expected to further influence the distribution and epidemiology of orbiviruses. Rising temperatures and altered precipitation patterns are predicted to expand the geographic range of *Culicoides* vectors, extend their active seasons, and increase the availability of suitable breeding habitats (3, 8). For example, higher mean annual temperatures and age were correlated with increased BTV seroprevalence in water buffalo and cattle in southern Italy (50). These environmental shifts may promote viral transmission dynamics, particularly in landscapes with high host densities and modified habitats like wastewater lagoons and fragmented agricultural areas (1).

Although logistic regression modeling in the present study did not find significant associations in weather or herd demographics and BTV/EHDV status, the small sample size likely limited the power of the analyses. Additionally, the cross-sectional design of the study provides a snapshot of vector-host interactions but does not allow us to determine causality. Future research should include longitudinal studies to track seroconversion and PCR positivity over time, coupled with ecological assessments of vector populations and habitats. Collaborations between wildlife and livestock health sectors are essential for integrating surveillance within a One Health framework.

Overall, our findings reveal significant age-associated dynamics in the epidemiology of BTV and EHDV in North American bison, with older animals showing higher seroprevalence but reduced PCR positivity. This suggests that immunity to orbiviruses accumulates over time due to repeated exposures, while risk of detectable viremia declines with age. Long term persistence of antibodies to both BTV and EHDV serotypes have been noted, supporting the concept that immunity following infection is generally long lasting for that specific serotype (51, 52). Additionally, the timing of sampling within the vector season may influence observed seroprevalence and viremia rates, as animals sampled at the end of the season may have had more opportunities for exposure and antibody development, while those sampled earlier may be more likely to exhibit active infection. Nonetheless, these dynamics highlight the importance of considering life stage in disease surveillance and management strategies. Younger bison, which were more likely to exhibit active infections in our study, may contribute disproportionately to virus transmission during peak vector activity, while older individuals may serve as immunological sentinels, reflecting historical exposure to these viruses. To better understand these patterns, longitudinal sampling across multiple seasons to capture variations in exposure, immune response, and viremia over time is advised.

Understanding these age-related patterns is critical for designing targeted surveillance programs and control measures. Future research should explore how these dynamics interact with environmental and vector-related factors to influence disease transmission at the wildlife-livestock interface. Enhanced surveillance efforts that incorporate bison as a model for understanding *Orbivirus* ecology can provide valuable insights into mitigating risks to livestock, wildlife, and ecosystem health. These results contribute to the growing body of evidence supporting the importance of wildlife in *Orbivirus* dynamics, particularly under the influence of climate change.

## Data Availability

The original contributions presented in the study are included in the article/supplementary material, further inquiries can be directed to the corresponding author.
